# LocateP: Genome-scale subcellular-location predictor for bacterial proteins

**DOI:** 10.1186/1471-2105-9-173

**Published:** 2008-03-27

**Authors:** Miaomiao Zhou, Jos Boekhorst, Christof Francke, Roland J Siezen

**Affiliations:** 1 Centre for Molecular and Biomolecular Informatics, Radboud University Nijmegen Medical Centre, PO Box 9101, 6500 HB Nijmegen, The Netherlands; 2 TI Food and Nutrition, and Kluyver Centre for Genomics of Industrial Fermentation, Wageningen, The Netherlands; 3 NIZO Food Research, Ede, The Netherlands; 4 Department of Biology, Faculty of Science, Utrecht University, Utrecht, The Netherlands

## Abstract

**Background:**

In the past decades, various protein subcellular-location (SCL) predictors have been developed. Most of these predictors, like TMHMM 2.0, SignalP 3.0, PrediSi and Phobius, aim at the identification of one or a few SCLs, whereas others such as CELLO and Psortb.v.2.0 aim at a broader classification. Although these tools and pipelines can achieve a high precision in the accurate prediction of signal peptides and transmembrane helices, they have a much lower accuracy when other sequence characteristics are concerned. For instance, it proved notoriously difficult to identify the fate of proteins carrying a putative type I signal peptidase (SPIase) cleavage site, as many of those proteins are retained in the cell membrane as N-terminally anchored membrane proteins. Moreover, most of the SCL classifiers are based on the classification of the Swiss-Prot database and consequently inherited the inconsistency of that SCL classification. As accurate and detailed SCL prediction on a genome scale is highly desired by experimental researchers, we decided to construct a new SCL prediction pipeline: LocateP.

**Results:**

LocateP combines many of the existing high-precision SCL identifiers with our own newly developed identifiers for specific SCLs. The LocateP pipeline was designed such that it mimics protein targeting and secretion processes. It distinguishes 7 different SCLs within Gram-positive bacteria: intracellular, multi-transmembrane, N-terminally membrane anchored, C-terminally membrane anchored, lipid-anchored, LPxTG-type cell-wall anchored, and secreted/released proteins. Moreover, it distinguishes pathways for Sec- or Tat-dependent secretion and alternative secretion of bacteriocin-like proteins. The pipeline was tested on data sets extracted from literature, including experimental proteomics studies. The tests showed that LocateP performs as well as, or even slightly better than other SCL predictors for some locations and outperforms current tools especially where the N-terminally anchored and the SPIase-cleaved secreted proteins are concerned. Overall, the accuracy of LocateP was always higher than 90%. LocateP was then used to predict the SCLs of all proteins encoded by completed Gram-positive bacterial genomes. The results are stored in the database LocateP-DB [1].

**Conclusion:**

LocateP is by far the most accurate and detailed protein SCL predictor for Gram-positive bacteria currently available.

## Background

**Figure 1:**
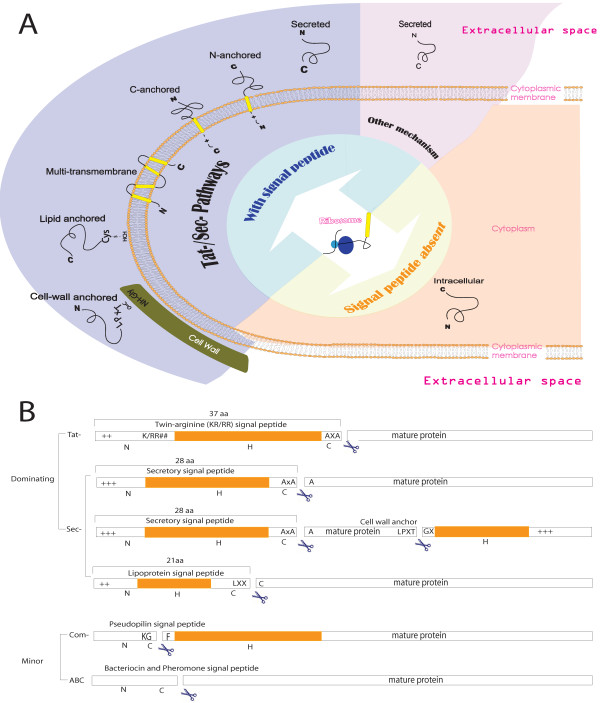
**(A): Classification of protein SCLs in Gram-positive bacteria**. The secreted proteins can be divided into the following subgroups: (i) N-terminal hydrophobic tail anchored (N-anchored), (ii) C-terminal hydrophobic tail anchored (C-anchored), (iii) covalent lipid-anchored, (iv) covalently/non-covalently cell-wall anchored, (v) secreted/released (defined as proteins that are Sec-/Tat-secreted and cleaved by the signal peptidase I), and (vi) non-classically secreted/released proteins *via *minor pathways [120, 163]. Based on the Swiss Prot classification system the SCLs could be categorized into: Cytoplasmic, Membrane (multi-transmembrane, N-/C-anchored), Cell wall (LPxTG-anchored) and Extracellular (lipid-anchored, secreted, bacteriocin-like) proteins. **(B): The structure of known signal peptides**. The overall structure of Tat- and Sec-dependent signal peptides is commonly conserved as distinct consecutive N, H and C regions. The N region is the start of the protein containing positively charged residues. The H region follows the N region and is a string of consecutive hydrophobic residues which can form an α-helix in the membrane. The C region contains the signal peptidase cleavage signals. Known cleavage/retention signals include the AxAA type I SPase cleavage site [163, 172], the L-x-x-C (so-called lipobox) type II SPase cleavage site [157] and the AxA Tat-substrate cleavage site [88, 90, 173]. The LPxTG-type motif is a C-terminal sorting signal which is involved in the covalent attachment of proteins to the peptidoglycan of the cell wall. The signal peptide of proteins targeted for minor secretion pathways does not follow the N-H-C structure [2, 125, 163].

In bacteria, secreted proteins are involved in stress sensing, substrate binding, cell communication, microbe-host interaction, adhesion, and other essential processes relevant to the environment and life style of the organisms. The secreted proteins are exported via various mechanisms and are retained by the bacterial cell via various interactions or released to the medium (Figure 1A). To identify the "secretome" [2] on a genome scale, subcellular proteomic studies have been carried out [3-7]. Although these experimental methods have contributed greatly to our knowledge of the subcellular location (SCL) of a variety of proteins, until now their scope has remained limited. In contrast, high-throughput computational methods for prediction of SCL sequence characteristics can be easily applied to every species whose genome has been sequenced.

Computational methods have gained considerable precision in the past decades. Initial tools focussed on detecting the presence, type and location of protein transmembrane segments, including signal peptides for targeting and translocation of proteins. One of the very first SCL prediction methods was introduced by Kyte and Doolittle [8] in 1982 with their amino acid hydropathy index. Since the late 90's machine-learning methods became more prominent, including neural networks [9-11], hidden Markov models (HMM) [12-14], support vector machines [2,15-23], Bayesian networks [24,25], and combined algorithms [26-33]. Moreover, present studies tend to combine different resources and methods [34-37]. For example, Chou *et al*. [38] combined gene ontology and functional domain databases, Shatkay *et al*. [39] combined text search and sequence data, and Marcotte *et al*. [40] combined protein homology and phylogenetic profiles in their studies.

Unfortunately, as a result of the trade-off between specificity and accuracy, computational methods will always be prone to error. Moreover, the number of false predictions increases even further when the SCL-related sequence characteristics have not been properly identified. For instance, among the Sec-dependent exported proteins, current predictors have severe difficulties to distinguish the proteins that are cleaved from the cell membrane by the type I signal peptidase (SPIase) – in this paper we will refer to these proteins as "secreted" – from a relatively large group of membrane-anchored proteins that also contain a putative SPIase-cleavage site but are not cleaved – in this paper we will lump these proteins in the category "N-anchored" [41-43].

As knowledge on the precise SCL of a protein is especially important to judge the biological nature and role of its activity, we constructed a new SCL prediction pipeline called LocateP. Our pipeline is geared to identify the detailed SCL of bacterial proteins by combining existing and novel prediction tools. Special effort was made to increase the accuracy of the prediction of N-anchored proteins. The version of LocateP presented here focuses on SCL prediction of proteins from Gram-positive bacteria.

## Results

### The construction of the SCL-prediction pipeline LocateP

A major drawback of most current sub-cellular location (SCL) predictors is that they are not aimed at the prediction of very specific SCLs but merely at the rather broad locations intracellular, membrane bound/associated and extracellular, in line with the Swiss Prot classification system. We therefore constructed a SCL predictor pipeline LocateP, that distinguishes 7 SCLs and 3 targeting pathways that can be identified in Gram-positive bacteria, with a focus on extracellular SCLs (see Figure 1A).

**Figure 2:**
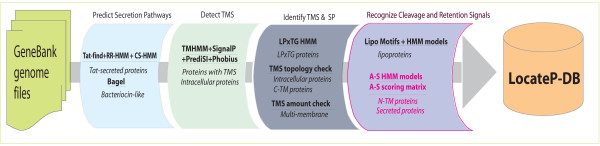
**Flowchart of the LocateP pipeline**. Firstly, the possibility of being secreted by the Tat pathway was calculated by combining Tat-find v1.2 [91] and our Tat-specific HMMs (RR-HMM, CS-HMM). Bacteriocin-like proteins were identified using Bagel [149]. Secondly, Phobius [14], PrediSi [98], SignalP 3.0 [18] and TMHMM 2.0 [12] were combined to identify transmembrane regions. Those proteins without any predicted TM segments were considered intracellular, whereas those with TM segments were divided into multi-TM membrane proteins, N-anchored membrane proteins or secreted/released proteins (single N-terminal TM segment, possibly signal peptide), and C-anchored membrane proteins (signal peptide and single C-terminal TM segment). Thirdly, a sortase-substrate HMM [165] was used to distinguish LPxTG-type peptidoglycan-anchored proteins from C-anchored membrane proteins. Subsequently, signal peptidase type II (SPII) substrates were predicted by combining existing lipoprotein motif models [41, 157] and new lipoprotein HMMs. The remaining proteins were classified into the categories secreted/released or N-anchored membrane proteins. See Methods and additional file 1 for more details. Abbreviation: A-S = Anchored-Secreted; TMS = TransMembrane Segment; SP = Signal Peptide; C/N-TM = C/N-terminally transmembrane anchored; LPxTG = LPxTG cell-wall anchored.

The LocateP pipeline was designed such that it mimics the protein secretion process in Gram-positive bacteria. The pipeline structure can be categorized as follows: (1) secretion pathway prediction, (2) transmembrane-segment detection, (3) signal peptide identification, and (4) cleavage and retention signal recognition. The LocateP pipeline employs existing SCL prediction tools (Table 1) as well as our own new and more accurate methods for the prediction of lipoproteins, Tat-secreted, N-terminally anchored, C-terminally anchored and secreted proteins (see Methods). LocateP uses at least 2 prediction methods for each SCL, in order to increase prediction accuracy. The selection criteria imposed on these methods were derived from literature. The LocateP pipeline is depicted in Figure 2; its construction is described in more detail in the "Methods" section and in the legend of Figure 2. A detailed flow chart is presented in Additional file 1.

**Table 1:** Recent methods for protein SCL prediction

**Speciality**		**Tool**	**Reference**
Membrane protein predictor	a	TMHMM	[12]
Both transmembrane helices and signal peptide predictor	a	Phobius	[14]
Signal peptide predictor	a	SignalP	[18]
	a	Predisi	[98]
		Signal peptidase type I cleavage site motif	[41]
Lipoprotein predictor	b	LipoP	[151]
	a	Signal peptidase type II cleavage site motif	[41, 157]
Tat-secreted protein predictor	b	TatP	[86]
	a	Tat-find.v.1.2	[174]
Protein subcellular location classifier	b	Psortb.v.2.0	[17]
	b	CELLO	[20]
	b	Gpos-PLoc	[28]
		Augur	[27]
Minor pathway secreted protein predictor	a	Bagel	[149]
		SecretomeP 2.0	[128]
Mycobacteria protein SCL predictor	b	TBpred	[95]

a, Tools included in the LocateP pipeline

b, Tools used for comparison and validation of LocateP

### Making the distinction between N-anchored and secreted proteins containing a SPI-cleavage site

In the past, the sequence corresponding to the signal peptide has been subdivided into three distinct regions: the N, H and C regions [28,44-46] (Figure 1B). Most of the membrane proteins with a single N-terminal TM anchor are easily identified as they do not have a predicted cleavage site for signal peptidases. However, as mentioned above, the prediction of SCL of proteins containing a putative signal peptidase type I (SPIase) cleavage site appears particularly difficult for current SCL predictors. Although many Sec-exported proteins are cleaved by the SPIase, a considerable number of proteins is not cleaved and remains membrane-anchored via the N-terminus [41].

**Figure 3:**
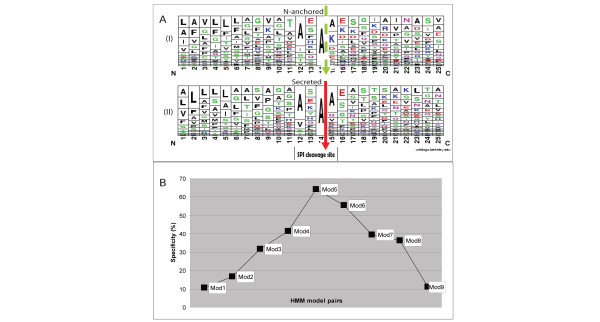
**Distinguishing between secreted and N-anchored proteins**. Tjalsma *et al*. [41] have identified 33 N-anchored and 36 secreted proteins from *Bacillus subtilis *(by 2D gel electrophoresis) which have a putative SPI-cleavage site motif in the C-region that follows the transmembrane helix H-region (see Fig. 1B). **(A): **A sequence composition chart, made using WebLogo [47], based on multiple-sequence alignment of the H- and C-regions (see Fig. 1B) of the N-anchored and secreted protein sets. The red arrow indicates the cleavage position of true SPI-site motifs (see Figure 1B), and the green dashed arrow represents the corresponding position in N-anchored proteins that is not cleaved. **(B): **The specificity of HMMs of different lengths containing the putative cleavage site A* = the Alanine after which cleavage takes place. Mod1: residues -9 to A*; Mod2: residues -11 to A*; Mod3: residues -14 to A*; Mod4: residues -8 to +3 of A*; Mod5: residues -13 to +10 of A*; Mod6: residues -8 to +17 of A*; Mod7: residues -3 to +10 of A*; Mod8: residues -3 to +17 of A*; Mod9: residues +1 to +25.

To identify the features that determine cleavage, the multiple sequence alignments of the signal peptides from experimentally validated N-anchored and secreted proteins [41] containing a putative SPI cleavage site in *Bacillus subtilis *were analyzed. To enhance the signal, orthologous sequences from other *Bacilli *were added in the analysis (see Materials and Methods). The Weblogos [47] of the two collections of sequences are given in Figure 3A. No distinguishing pattern could be detected by eye. Therefore, a series of HMMs were constructed based on the sequence alignments of the N-anchored and secreted proteins. Nine pairs of HMMs were built for sequences surrounding the putative SPI cleavage site. Different numbers of residues on either side of the putative cleavage site were included in the models in order to investigate the roles of the H-region and the C-region in cleavage-site recognition. When the HMM pairs were applied to the two respective sets of sequences, it appeared that the HMM pair containing an equal number of residues on either side of the putative cleavage site performed best in predicting correctly whether the cleavage site was genuine or not (Figure 3B). The individual HMMs were not mutually exclusive for either of the two sets of sequences. However, when the scoring of the two HMMs of the pair was combined into a scoring matrix, the experimentally determined non-cleaved and cleaved sequences could be distinguished almost perfectly. The scoring matrix was included in LocateP; details of the matrix are described in the legend of Figure 3 and in the "Methods" section.

### Initial validation of LocateP

Ideally, the performance of the LocateP pipeline should be checked with large experimentally validated data sets. Unfortunately, the availability of such large data sets is rather limited. LocateP was tested first with the experimental data set of Tjalsma *et al*. [41] which was used to create the HMM pair that distinguishes the N-anchored and secreted proteins containing a putative SPI-cleavage site. LocateP was able to distinguish these proteins with an accuracy of >90%. Then the performance of LocateP was tested on ten other data sets. These sets were extracted from literature describing other SCL prediction tools. LocateP performed extremely well on these sets, as the prediction accuracy was always higher than 90% (Table 2).

**Table 2:** Comparison of the performance of LocateP with other SCL prediction tools. The entry in each cell indicates the recall of the method with respect to the data in the test-set (TS). * indicates that the test data were extracted from experimental studies. N/A indicates that a certain tool was not applied to the test sets because that set could not be treated appropriately by the tool. The size of the test sets (TS) is indicated in brackets and the relevant literature is mentioned in the Table legend.

**Comparison of LocateP and other SCL prediction tools**								
**Methods**	**TS 1 (171)**	**TS 2 (1077)**	**TS 3 (236)**	**TS 4 (36)**	**TS 5 (78)**	**TS 6 (43)**	**TS 7 (47)**	**TS 8 (103)**

LocateP	98.8%	99.4%	97.5%	97.2%	91.0%	95.7%	97.9%	98.1%
LipoP	N/A	96.8%		N/A	N/A	89.4%	95.7%	N/A
SignalP 3.0-NN	99.3%		98.3%	97.2%	25.6%	N/A	N/A	N/A
SignalP 3.0-HMM	99.4%		96.6%	97.2%	20.5%	N/A	N/A	N/A
Phobius	98.8%		96.6%	97.2%	42.3%	N/A	N/A	96.1%
Predisi	99.4%		93.2%	94.4%	37.2%	N/A	N/A	N/A
TMHMM	N/A	99.3%	N/A	N/A	N/A	N/A	N/A	97.1%

Psortb v.2.0	N/A	N/A	49.2%	36.1%	10.3% (M) 1.3% (E)	18.6% (M) 4.7% (E)	10.6% (M) 4.3% (E)	N/A
Cello	N/A	N/A	82.6%	80.6%	75.6% (M) 8.0% (E)	61.7% (M) 16.3% (E)	68.1% (M) 27.7% (E)	N/A

**Comparison of LocateP, Tat-find v1.2 and TatP in the prediction of Tat-secreted proteins**								

**Methods**	**TS 3+ TS 4 (272)**			**TS 9a (713)**		**TS 9b (632)**		

TatP	92.8%			99.6%		96.5%		
Tat-find v1.2	94.9%			98.6%		93%		
LocateP	93.6%			99.9%		98.4%		

**Comparison of LocateP, Cello and Psortb v2.0 based on data sets extracted from Swiss-Prot**								

**Methods**	**TS10a (196)**	**TS10b(129)**	**TS10c(108)**	**TS10d(14)**	**TS11a (340)**	**TS11b(60)**	**TS11c(402)**	**TS11d(50)**

LocateP	98%	97%	80.6%^e^	84%^f^	97.4%	96.7%	86.1%	86%^g^
Psortb v.2.0	93.9%	91.7%	79.6%	50%	89.1%	6.7%	81.1%	80%
CELLO^d^	97%	99.2%	97.2%	57.1%	94.1%	56.7% (E) 43.3% (M)	87.6%	94%
TBPred^h^	N/A	N/A	N/A	N/A	94.71%	68.33%	87.81%	50%

The test sets are: TS1 [175], TS2 [98]^NGP ^= Cytoplasmic; TS3 [98]^PGP^, TS4 [41]* = Secreted; TS5 [41]*^a ^= N-anchored; TS6 [157]*, TS7 [151]^c ^[41]^b^* = Lipid-anchored; TS8 [175] = Membrane; TS9a [86]^TestRR ^= Cytoplasmic; TS9b [86]^TestRR ^= Membrane; TS 10a [28]^Test,Training ^= Cytoplasmic; TS 10b [28]^Test,Training ^= Membrane; TS 10c [28]^Test,Training ^= Extracellular; TS 10d [28]^Test,Training ^= Cell wall; TS11a [95]^Training ^= Cytoplasmic; TS11b [95]^Training ^= Lipid-anchored; TS11c [95]^Training ^= Membrane; TS11d [95]^Training ^= Secreted.

Abbreviations: TS: test set; M: Membrane; E: Extracellular; Test: test set of this article; Training: training set of this article; NGP: negative training set containing only Gram-positive bacterial proteins; PGP: positive training set containing only Gram-positive bacterial proteins; RR: the proteins contain twin-arginine residues in the initial 35 residues.

^a^: 30 proteins of this set contained putative SPI-cleavage site and were included in LocateP training process

^b^: After removing redundancy, 47 proteins were left in this set

^c^: The set contains both Gram-positive and Gram-negative bacterial proteins

^d^: Only the predictions with highest score were taken

^e^: 17 proteins in this test set were either proven to be secreted or they were found to be secreted via minor secretion pathways. LocateP focuses on the prediction of major secretion systems, therefore these proteins were predicted as "intracellular", which meant that no classical signal peptides were found in these proteins.

^f^: Most of the proteins in this set are associated on the cell wall via non-covalent interactions such as protein-protein interaction.

^g^: 23 out of 50 proteins in this set were predicted as "N-anchored" proteins by LocateP, indicating that these proteins could be secreted via Sec-pathway but remained attached to the cytoplasmic membrane of the cell.

^h^: among the support-vector machines involved in TBPred only the best performance with the appropriate protein class was taken.

A second check was done with data collected from TransportDB [48]. Based on expert knowledge on the composition and location of various transport systems and their functional components, the SCL prediction of 1336 transport-related proteins from *Bacillus subtilis *168, *Bacillus cereus *ATCC14579 and *Lactobacillus plantarum *WCFS1 was verified (Table 3). For a difficult group like the substrate-binding proteins of ABC transport systems, LocateP identified 113 of 124 proteins in a correct SCL for substrate binding (96 lipoproteins, 6 secreted and 11 N-anchored proteins) [49-59]. For the other groups of transport-related proteins, the predicted SCL fitted the biological role of the transport proteins in ~98% of the cases.

**Table 3:** Validation of LocateP predictions of transporter systems using the annotation in TransportDB

**Species**	**Number of transport-related proteins with identified SCL**	**LocateP accuracy**
*Bacillus subtilis *168	426	98.2%
*Bacillus cereus *ATCC14579	571	97.5%
*Lactobacillus plantarum *WCFS1	373	98.8%

A third less quantitative check included a comparison of the LocateP predictions for all N-anchored and secreted proteins of the *Bacillus subtilis *genome with their NCBI functional annotations (Additional file 2, 3 and 4). Nearly all of the predictions appeared to make biological sense according to literature: most of the predicted N-anchored proteins were annotated to be involved in processes that are related to the cell-envelope, such as cell division, transport, cell-envelope biogenesis, mobility, competence, signal transduction, protein turnover, etc; most predicted secreted proteins were indeed known to be secreted enzymes such as extracellular carbohydrases [60], alkaline phosphatases [61,62], metalloproteases [63], neutral proteases, and subtilisin-family proteases [64].

### Further validation and comparison of LocateP with other tools and pipelines

Recently, Gardy *et al*. [44] have compared most of the current SCL classifiers, and some tools showed excellent performance. We compared the performance of LocateP to a selection of these tools, including the individual SCL predictors and other general integrative SCL classifiers that were considered best (Table 2).

#### On N-anchored, secreted, lipid-anchored and multi-transmembrane protein prediction

LocateP and several individual SCL prediction tools were applied to the same collection of reference data sets. LocateP showed similar or higher recall to PrediSi, Phobius and SignalP 3.0 at signal peptide detection, respectively (Table 2, test sets 1, 2, 3, 4). LocateP performed clearly better than all other tools at predicting lipoproteins and multi-transmembrane proteins (Table 2, test sets 6, 7, 8). For the group of N-anchored proteins, LocateP clearly outperformed all other tools with a much higher prediction specificity and accuracy (Table 2, test set 5).

As has been noted by others [12,14,42], the N-anchored membrane proteins form an ambiguous group with respect to the location of their biological activity, i.e. outside or inside the cell. Various N-anchored proteins are actually active at the cytoplasmic side of the bacterial cell membrane [65-75]. Due to the lack of reliable distinguishing algorithms and experimental data, no reliable prediction methods for these "outside-in" proteins are available yet [12,29,43,76-78]. As a result, in the current version of LocateP, proteins are only annotated as "N-terminally anchored"; most are presumed to function outside the cell, while some might have intracellular activity. A few of the known intracellular cases are indicated in Additional file 2.

#### On Tat-secreted protein prediction

Recent research pointed out that the Tat-export pathway plays an important role as a parallel protein secretion pathway to the Sec-pathway in some Gram-positive organisms [79-85]. Unfortunately, Sec-signal peptide detectors have a high false-negative prediction rate on Tat-substrates [86]. Therefore, we considered it necessary to include a Tat-secreted protein prediction tool in the LocateP pipeline, and we combined two newly created Tat-secreted protein-specific HMMs (see Methods) with Tat-find v.1.2 for the SCL prediction of these proteins. Tat-signal peptides are known to have an almost invariable double Arg or Lys+Arg motif (RR-motif) [87-90] upstream of the transmembrane segment. It appeared important that the Tat-secreted protein predictors can discriminate the Tat-signal peptides from sequences (especially transmembrane helices) that contain consecutive positively charged residues.

We compared the performance of LocateP, TatP and Tat-find v1.2 on the proteins containing a RR/RK pattern in their N-terminus (test sets 3, 4 and 9). LocateP clearly performed better than the other two specific tools when tested with intracellular and membrane proteins sets, and thus showed an excellent capability of Tat-signal peptide detection (Table 2). Moreover, it appeared that TatP and Tat-find v1.2 predicted several proteins to be secreted via the Tat-pathway in 22 species that apparently lack the relevant pathway genes [91], whereas LocateP did not find any Tat-pathway substrates in those species. Thus, LocateP showed the best overall performance among the Tat-pathway prediction tools for gram positive bacteria.

#### Comparing LocateP and other integrative SCL classifiers

According to the comparative study of Gardy *et al*. [44], CELLO [20] is one of the best SCL classification pipelines. We therefore evaluated the performance of LocateP as an integrative SCL classifier by comparing it to CELLO and the widely used pipeline Psortb.v.2.0 [25]. Other pipelines like SubLoc [92], LOCtree [93], Proteome Analyst [94], P-CLASSIFIER [33] and PSLpred [36] were not selected because they either do not provide prediction of membrane proteins, or are tailored for Gram-negative bacteria, or in the best case showed similar performance to Psortb.v.2.0 or CELLO. Recently, a SCL prediction tool called Gpos-Ploc [28] was published that classifies Gram-positive proteins. LocateP was not compared to Gpos-Ploc because its web server accepts only one sequence per search. Moreover, the overall accuracy of the tool is reported to be only ~85% [28].

LocateP had an accuracy lower than CELLO (Table 2) when tested with data extracted from the Swiss-Prot database (test set 10 [28]). However, when compared using experimental data (test sets 3, 4, 5, 6, 7), CELLO and Psortb v2.0 showed dramatically poor prediction rates (Table 2). This poor performance relates to the fact that the training data of CELLO and Psortb v.2.0 were from the Swiss-Prot database (i.e., part of test set 10 and 11). This database does not distinguish between N-anchored, secreted and lipoproteins, and at the same time the members of these groups are distributed over two general classes: "membrane" and "extracellular". Thus, in essence the poor performance of CELLO and Psortv.b.2.0 is a consequence of the less-specific classification in Swiss-Prot (Table 2). *Vice versa*, the lower accuracy of LocateP on the Swiss-Prot data is related to the inconsistency in the classification.

TBPred [95] is a SCL classifier that was especially designed for mycobacteria, based on the idea that organism-specific methods might have higher accuracy [96,97]. We compared LocateP with TBPred using the training data of TBPred (test set 11). Surprisingly, LocateP showed considerably higher accuracy than TBPred, especially on lipoprotein and secreted protein prediction, even though no mycobacterial proteins were involved in the lipoprotein prediction training process of LocateP.

Finally, the performance of LocateP was compared to Augur [27], a computational pipeline that also combines many existing tools. Augur detects signal peptides and transmembrane helices using only SignalP and TMHMM, and consequently the accuracy of N-anchored protein prediction of Augur is much lower than with LocateP. Augur also falsely predicted 8 lipoproteins out of a test-set of 114 non-lipoproteins (test sets 4 and 5), which implied a higher false-positive rate than LocateP on lipoprotein prediction.

### Comparative analysis of protein subcellular location in Gram-positive bacteria

LocateP was applied to the encoded proteins of all complete Gram-positive bacterial genomes available in the NCBI database. The average distribution of proteins grouped by predicted SCL was calculated for each genome. Despite the different genome sizes, Gram-positive bacteria tend to have a similar distribution of proteins over certain SCLs independent of class or family, and this independency also holds for individual Gram-positive bacterial genomes (Table 4). We note that the fractions of intracellular and membrane proteins predicted by LocateP in Gram-positive genomes were consistent with what was previously estimated by other tools [12,18,24,25]. The complete genome predictions can be viewed in our database LocateP-DB [1].

**Table 4:** LocateP-predicted average distribution (%/(STDEV)) of proteins over different SCLs for Gram-positive bacteria

**Class/order level**								
Species	Actinobacteria	Bacillales	Clostridia	Lactobacillales	Mollicutes			

Average genome size	4098	3573	2969	2048	724			

**Grouped according to LocateP classification**								

N-anchored (Membrane)	5.0/(1.1)	5.7/(0.6)	6.8/(1.0)	5.8/(0.7)	8.7/(3.1)			
C-anchored (Membrane)	0.3/(0.2)	0.1/(0.1)	0.2/(0.1)	0.2/(0.1)	0.3/(0.3)			
Multi-transmembrane (Membrane)	16.5/(2.6)	20.3/(1.4)	16.9/(2.8)	17.9/(2.1)	17.1/(2.3)			
Intracellular (Cytoplasmic)	74.3/(2.8)	69.8/(2.2)	73.2/(3.6)	72.9/(2.0)	71.4/(3.8)			
Lipid anchored (Extracellular)	2.2/(0.5)	2.3/(0.4)	1.6/(0.6)	1.6/(0.5)	1.9/(1.6)			
Secreted (Extracellular)	3.0/(0.9)	2.1/(0.5)	2.1/(0.5)	1.8/(0.6)	2.3/(1.3)			
Secreted via minor pathways (Extracellular)	0.1/(0.1)	0.1/(0.1)	0.1/(0.1)	0.28/(0.2)	0.04/(0.1)			
LPxTG Cell-wall anchored (Cell wall)	0.1/(0.2)	0.4/(0.4)	0.1/(0.2)	0.6/(0.4)	0.03/(0.1)			

**Grouped according to Swiss-Prot classification**								

Membrane	21.4/(2.7)	26.2/(1.7)	23.8/(3.4)	23.8/(1.9)	26.1/(3.9)			
Cytoplasmic	74.3/(2.8)	69.8/(2.2)	73.2/(3.6)	72.9/(2.0)	71.4/(3.7)			
Extracellular	5.4/(1.1)	4.5/(0.7)	3.8/(0.8)	3.7/(0.8)	4.2/(1.9)			
Cell wall	0.1/(0.2)	0.4/(0.4)	0.1/(0.2)	0.6/(0.4)	0.03/(0.1)			

**Species level**								

Organism	*Spn*	*Lla*	*Sau*	*Lmo*	*Lpl*	*Cac*	*Bsu*	STDEV

Total proteins	2105	2321	2656	2846	3009	3672	4105	

**Grouped according to LocateP classification (%)**								

N-anchored (Membrane)	4.5	5.9	6.0	4.9	5.2	6.9	6.2	0.8
C-anchored (Membrane)	0.1	0.1	0.1	0.4	0.2	0.2	0.1	0.1
Multi-transmembrane (Membrane)	17.9	18.4	19.5	19.1	20.5	18.1	20.7	1.1
Intracellular (Cytoplasmic)	74.7	72.8	70.5	71.1	70.2	71.3	69.1	1.9
Lipid anchored (Extracellular)	1.7	1.4	2.2	2.0	1.6	1.7	2.0	0.3
Secreted (Extracellular)	1.2	1.9	2.1	1.7	1.9	2.3	2.6	0.4
Secreted via minor pathways (Extracellular)	0.5	0.0	0.1	0.2	0.3	0.1	0.2	0.2
LPxTG cell-wall anchored (Cell wall)	0.5	0.5	0.5	1.5	1.1	0.1	0.1	0.5

**Grouped according to Swiss-Prot classification (%)**								

Membrane	22.4	24.4	25.5	24.4	25.9	25.2	27.0	1.4
Cytoplasmic	74.7	72.8	70.5	71.1	70.2	71.3	69.1	1.9
Extracellular	3.4	3.3	4.4	4.0	3.8	4.1	4.8	0.5
Cell wall	0.5	0.5	0.5	1.5	1.1	0.1	0.1	0.5

Abbreviations: *Spn*: *S. pneumoniae*; *Lla*: *L. lactis*; *Sau*: *S. aureus*; *Lmo*: *L. monocytogenes*; *Lpl*: *L. plantarum*; *Cac*: *C. acetobutylicum*; *Bsu*: *Bacillus subtilis*

## Discussion

Although the early SCL-prediction tools performed rather poorly, current tools perform rather well on specific categories of signal-peptide containing proteins and membrane proteins [44], reaching an accuracy of 96%. Nevertheless, for other groups like secreted, N-anchored and lipoproteins these tools still perform rather poorly. As the latter groups represent a considerable part of the secretome, we decided to design a new SCL-identification pipeline called LocateP.

The performance of LocateP was checked against the best current tools and it outperformed all of them, particularly when difficult groups of proteins and SCLs were concerned. The outstanding performance was achieved though the generation of specific HMMs based on protein sequences whose cellular fate had been experimentally tested. For instance, it has long been a problem to identify secreted and N-anchored proteins from the group of proteins carrying a putative SPI-cleavage site motif. Formerly, the H-region together with the cleavage site were considered to be the key elements of SPIase-substrate recognition. Therefore, previous signal-peptide predictors were constructed focusing on the H-region and/or on the cleavage site [14,18,26,98-100]. However, Carlos *et al*. [101] found that the H-region of the SPIase substrate was not critical for peptidase-cleavage capability but that, in contrast, mutations in the C-region of originally non-cleaved proteins caused alternative cleavage. They therefore claimed that specific substrate-enzyme interactions around the C-region should be decisive for SPIase-cleavage site recognition. Indeed, our analysis of the signal sequences of a group of secreted and N-anchored proteins indicated that the C-region is important, but that at the same time also the H-region carries properties that determine the protein's fate (i.e. to be or not to be cleaved). The fact that the performance of the dedicated HMMs became worse when the sequence was extended beyond 30 residues implies that the decisive information is present in this stretch of sequence. LocateP improved the separation of N-anchored and secreted proteins from ~40% (by Phobius [41]) to > 90% without disturbing the SCL prediction of the other types of proteins.

LocateP was designed as a pipeline, and hence could have performed less well on specific categories than specialized tools. In particular, the performance would have been considerably lower if the flow scheme had been chosen wrongly. However, a comparison of the performance on lipoproteins, membrane proteins, Sec-secreted and Tat-secreted proteins with the specialized tools LipoP 1.0, TMHMM 2.0, Phobius, SignalP 3.0, Predisi, Tat-find v.1.2 and TatP shows that LocateP does not suffer from being a pipeline tool. Apparently, our choice to mimic the order in the bacterial secretion process was a correct one. In fact, it has been shown by others that the SCL prediction can be improved considerably by simulating the protein sorting processes [93,102]. Overall, LocateP performed very well, with an accuracy higher than 95% for nearly all categories, and only slightly lower in one case (91% for N-anchored proteins), but still considerably better than all other tools. LocateP could be used to distinguish 7 SCLs and 3 sorting pathways and avoided the inconsistent SCL classification which most SCL classifiers inevitably inherited from Swiss-Prot.

Because of the high prediction accuracy of LocateP on proteins of known biological function (see e.g. Additional file 2), we expect that the SCL prediction of proteins of unknown function should also be equally reliable. In principle, the genome-scale SCL predictions made by LocateP provide an excellent starting point for functional annotation and experimental analysis of encoded proteins of unknown function, as they provide numerous clues about where to look for a certain biological activity.

Although LocateP already performs quite well, there is inevitably room for improvement. For instance, in the Swiss-Prot database, many of the annotated cell-wall proteins are secreted proteins bound to the cell surface via non-covalent interactions. Known elements of non-covalent binding include choline-binding domains, LysM domains, type 2 cell-wall binding domains, GW-modules, Lysin-binding motifs, ChW-binding motifs, WxL domains, LPP-region binding, S-layer proteins, and others [103-117]. The current version of LocateP was designed to predict only the covalent cell-wall (peptidoglycan) binding mechanism of proteins by dedicated sortases. For instance, among the 14 cell-wall proteins in test set 10d [28], 13 are non-covalently cell-wall bound secreted proteins. LocateP correctly predicted 10 of these as "secreted proteins", but does not allow for the fact that these proteins could be cell-wall bound via non-covalent mechanisms after secretion (Table 2). Future versions of LocateP will include non-covalent binding to the cell wall.

Not all mechanisms of protein secretion or modification are known to date and not all have been included in the LocateP pipeline yet [118-123]. This is the case for proteins that have been shown to occur at various locations or those that are secreted via minor pathways. Examples are proteins that are either cleaved multi-domain proteins [124], auto-transporters found in both cytoplasmic and extracellular locations [125], or proteins with various SCL depending on growth phase and/or specific environment [124,126]. The multi-compartment proteins and minor-pathway secreted proteins appear to be rare in most bacteria, and their sorting mechanisms are not completely understood yet. Therefore, the current version of LocateP predicts only one SCL for such proteins, which may be only partially correct. In contrast, Psortb v2.0 and CELLO were claimed to be capable of multi-location prediction [126,127]. Both tools employ machine-learning methods and the predicted multiple locations should represent a certain statistical significance even without large-scale experimental evidence. However, both tools inevitably generate a considerable number of false positives. Similarly, SecretomeP 2.0 [128], which was made to predict non-classically secreted proteins, was not included in LocateP because of its high false-prediction rate. The recent predictors Euk-mPloc [129] and Hum-mPloc [97] incorporated up-to-date Eukaryotic proteins that were found to have multiple compartments and the tools achieved rather satisfying accuracies. Similar tools will be included or constructed for LocateP when more experimental data on multiple locations of bacterial proteins are available.

Another group of proteins that is not treated separately by LocateP is the group that is exported by unknown mechanisms and is known as the Gram-positive periplasmic proteins [130-133]. Carlsson *et al*. [134] recently reported that in Gram-positive bacteria the secreted proteins could be directed to different extracellular regions including a periplasmic space. In fact, the prediction of a subcellular location "periplasmic" in Gram-positive was not included in any published SCL prediction tools for Gram-positive bacteria, except in Gpos-PLoc [28]. However, the Gpos-PLoc prediction algorithm was based on only 5 proteins which were extracted from the Swiss-Prot database. Indeed, among these 5 proteins, four were expressed in the *E. coli *periplasmic space, but no evidence exists that they are also expressed in the periplasm of a Gram-positive organism [135-138]. Moreover, one protein (P29166) was proven to be located inside the cell [139]. LocateP predicted correctly that 4 of the 5 proteins should be secreted and are located outside of the plasma membrane, while P29166 was predicted to be cytoplasmic, in line with the experimental evidence.

LocateP was first tailored for the SCL prediction of Gram-positive bacterial proteins; therefore prediction of the Gram-negative specific proteins, such as β-barrel membrane proteins, was not yet included in the pipeline. LocateP was compared to SigTree [140], a signal-peptide detector based on sets of experimentally verified *E. coli *proteins, using the same data set from *E. coli *(data not shown). LocateP showed slightly lower accuracy than SigTree did, which suggests that the sequence composition of signal peptides from Gram-positive and Gram-negative bacterial proteins could be different. Future versions of LocateP will be improved and extended to Gram-negative bacterial protein SCL prediction by incorporating Gram-negative specific subcellular-location prediction tools.

Finally, we must emphasize that in several cases an automatic SCL prediction of a protein will inevitably give an incorrect prediction using LocateP or any other tool: (i) when the start codon of a gene encoding a protein with a signal peptide has been wrongly identified (either too far upstream or too far downstream), (ii) when a frame shift in the open-reading frame leads to different fragments of encoded proteins, and (iii) when an intracellular protein contains a signal peptide-like hydrophobic helix near the N-terminus; in this case such helices generally fold into the interior of the globular protein [141-144].

## Conclusion

As detailed and accurate genome-scale SCL prediction of encoded proteins is highly desired by scientists in various biological research areas, numerous existing and newly developed tools were combined into one pipeline: LocateP. To date, LocateP is the most detailed protein SCL predictor for Gram-positive bacterial proteins among all tools that have been reported, in that it presently distinguishes 7 different SCLs and 3 sorting pathways, with focus on extracellular SCLs. Moreover, it is also the most accurate SCL predictor, especially on distinguishing N-anchored and secreted proteins. LocateP was applied on all completed Gram-positive bacterial genomes from the NCBI sequence database. The results are updated synchronously with Genbank updates and are publicly available via the database LocateP-DB [1]. The present version contains SCL predictions for 436,771 proteins in 148 genomes of Gram-positive bacteria. These genome-scale SCL predictions provide an excellent starting point for experimentalists to improve the functional annotation of proteins.

## Methods

### Sources of sequence information and location data

The genome sequences of Gram-positive bacteria were extracted from GenBank on May 30^th^, 2007 ("ORGANISM" annotation fields: 'Firmicutes' or 'Actinobacteria'), and were continuously updated since then. Protein sequences of *Bacillus *species were collected both from GenBank on April 1^st^, 2007 and from the ERGO database [145] on November 15^th^, 2006.

Eight different protein data sets of known subcellular location were selected from literature describing other tools and describing proteome studies (Table 2 legend). In order to check the performance of the LocateP pipeline, the SCL predictions were checked against an expert evaluation of the functional location of transport-related proteins from several Gram-positive bacterial genomes in TransportDB [48] on May 30^th^, 2007, and an expert evaluation against the protein function annotation as retrieved from GenBank on August, 10^th^, 2007.

### Sequence analysis and evaluation of performance

Multiple sequence alignments were built with MUSCLE [146]. HMMs were built with HMMER [147]. Wherever appropriate, HMMs of varying length and different regions of the aligned N-terminal sequences of proteins were made, and the HMM that performed best was selected. Performance was evaluated using the statistical measure recall (or sensitivity) which is the number of true positives divided by the sum of the true positives and the false negatives.

### Bioinformatics tools included in the LocateP pipeline

Many studies have compared and evaluated currently available transmembrane segment and signal peptide predictors [41,44,76,98-100,148]. Based on those studies and our own preliminary trials the following tools were selected to be included in our SCL prediction pipeline LocateP: TMHMM 2.0 [12], Phobius [14], SignalP 3.0 [18], PrediSi [98], and Bagel [149] (Table 1). Of these, TMHMM 2.0 and SignalP 3.0 are the most popular ones in the field; Phobius was selected for its high specificity on transmembrane segment identification; PrediSi was selected because it was trained with comparatively recent experimental data, and because it slightly outperformed SignalP 3.0 when applied to Gram-positive bacterial proteins [98]. We also included the predictor Bagel for non-classically secreted bacteriocin-like proteins [149]. The membrane protein predictor MemType-2L [150] includes topology prediction of N-anchored proteins but showed rather low accuracy with our experimental datasets; therefore this tool was not included in LocateP. Some other tools were not incorporated either because of a high false-prediction rate (e.g. HMMTOP [13] and SecretomeP 2.0 [128]), a low specificity for Gram-positive bacteria (e.g. LipoP 1.0 [151]), or simply the lack of stand-alone installable software packages (e.g. TatP [86], Signal-3L[152], Signal-CF[153] and Tat-pred [154]).

### Signal peptide detection

LocateP detects signal peptides by scanning the protein N-terminus, which was defined as the initial 60 amino acids of the protein, using SignalP 3.0, Phobius and PrediSi. Some proteins have a signal peptide shortly after these 60 amino acids. These proteins were predicted as "intracellular", but we added the extra remark of "TMH start AFTER 60" to the annotation indicating that these proteins could be secreted. No attempt was made to choose alternative start codons of incorrectly predicted start sites of ORFs.

### Specific HMMs to determine the SCL of proteins with a putative SPI-cleavage site

Recently, Tjalsma *et al*. have experimentally determined the SCL of a large number of *Bacillus subtilis *proteins [41]. The experimental set contained 66 proteins with a putative SPI-cleavage site. Of these 36 appeared to be cleaved and thus secreted, whereas 30 were shown to remain N-anchored. We named these sets "EXP-secreted" and "EXP-anchored", respectively, and used them to construct set-specific HMMs. To enhance the inherent signal, both sets were expanded by adding orthologous sequences from other Bacilli. First, homologs were searched with BLASTP [155] in the ERGO genome database [145] using full-length sequences. Only the three best BLAST hits were considered orthologs, when they also showed conserved gene context and functional annotation, high similarity and similar protein length. In this way, after removing orthologs containing identical N-terminal sequences, 27 secreted and 23 N-anchored orthologs could be added to the "EXP-" sets.

Pairs of HMMs were built to separate the group of proteins with a putative SPI cleavage-site into those that are cleaved (i.e. secreted/released) and those that are not (i.e. N-anchored). The sequences were aligned around the putative cleavage site and the length of the HMMs was varied (length >8). All HMM pairs were applied to both "EXP-" sets; the E-value was set at 10,000 to assure each protein gained an HMM score. For each pair the separation between truly cleaved and truly N-anchored proteins was analyzed and it appeared that the HMM pair containing equivalent amounts of H and C region residues achieved the highest specificity in distinguishing the two sets (see Figure 3B). The most specific HMM pair had a length of 25 amino acids and ran from residue -14 to +10 relative to the cleavage site Alanine (see Figure 3A).

The individual HMMs of the selected pair (HMM_non-cleaved_, HMM_cleaved_) each displayed a relatively high specificity, but this was increased significantly by combining the two HMMs. A generic scoring scheme was derived via the following procedure: i) The HMM scores were rounded to discrete integers and the score distribution for the EXP-anchored and EXP-secreted protein sets was used to determine a first cut-off. The discrete HMM scores related to the HMM_non-cleaved _ranged from -19 to +20 with all non-cleaved (i.e. N-anchored) proteins scoring higher then 3, those related to the HMM_cleaved _ranged from -29 to +20 with all cleaved (i.e. secreted) proteins scoring higher then 0. In fact, for both HMMs only in a small scoring range the two protein groups overlapped. Therefore, all sequences with a score ≤2 using the HMM_non-cleaved _were attributed the SCL: SEC-secreted, and those with a score ≤-1 using the HMM_cleaved _were attributed the SCL: N-anchored. ii) For those sequences that scored >2 with the HMM_non-cleaved _and >-1 with the HMM_cleaved_, the score with both models was compared. In case HMM_cleaved _> HMM_non-leaved _score, the sequence was considered SEC-secreted, whereas, in case HMM_non-cleaved _≥ HMM_cleaved _score, the sequence was considered N-anchored.

### The creation and selection of a specific HMM for lipoprotein prediction

The experimental data of Tjalsma *et al*. [41] indicated that at least 42 distinct proteins of *Bacillus subtilis *are lipoproteins. This set of proteins was taken and expanded with orthologs from 18 closely related *Bacillus *species using an Inparanoid [156] search for best bi-directional hits. After removing the sequences which contain identical initial 50 residues, 219 putative orthologous lipoproteins could be added. As all lipoproteins are anchored to the cell membrane by thioether linkage of the conserved lipobox cysteine to a diglyceride [41,56,151,157,158], the sequences were aligned around the lipobox. Eight HMMs were built based on different N-terminal regions from these proteins varying in length between 5 and 30 residues. Each HMM was applied to the original dataset of Tjalsma *et al*. and the performance was evaluated. The HMM with a length of 21 residues (-20 residues to the lipobox Cysteine) showed the highest specificity when the T-score was set to 3. Gaps were allowed in this model except in the region of the lipobox (residue -5 to the lipobox Cysteine)

### The creation and selection of a specific HMM for Tat-secreted protein prediction

The 105 putative Tat-secreted proteins (according to Swiss-Prot) from the TatP-positive training set [86] were taken as the initial set for generating Tat-specific HMMs. The sequences were aligned either around the double Arg or Lys+Arg motif (RR-motif) [87-90] upstream of the transmembrane helix or the putative AxA triplet cleavage site [86] predicted by TatP downstream of the transmembrane helix. Eleven HMMs with different lengths were generated. A combination of two HMMs was found to be most specific with the training data, together with the restriction of an E-value smaller than 10: one HMM contained 2 residues in front and 16 after the twin-arginine motif, and the other HMM contained 17 amino acids in front and 1 residue after the triplet cleavage site. Interestingly, these two HMMs partly overlapped each other by the transmembrane (H) region. According to Taylor *et al*. [154], the -3 to +7 residues surrounding the twin-arginine should be the most characteristic for Tat-secreted protein identification. This conclusion was reaffirmed by our HMM model. The current tools Tat-find v1.2 and TatP both focus solely on the twin-arginine motif and consecutive transmembrane helix detection. In the prediction of the Tat-secretion signal our HMMs were combined with the Tat-find v1.2 program (in a scoring matrix) and therefore more weight was given to the prediction of the twin-arginine motif and its following hydrophobic region as Tat identifiers. This combined method was tested with 22 independent experimentally verified Tat-secreted proteins (20 of them are from *E. coli *[159-162], while PhoD and YwbN were from *Bacillus subtilis *[163,164]). The SCL of 20 of these proteins was correctly identified by LocateP, including PhoD and YwbN (these 2 proteins were not in the HMM training set). Using this procedure the false prediction rate was significantly decreased compared to Tat-find v1.2 and TatP (Table 2). It was suggested that the Tat-pathway in Gram-positive bacteria is structurally different from Gram-negative bacteria [80,86,90,120,163,164]. Although the Tat-secreted prediction of LocateP outperformed current tools, this part of the tool was trained with Gram-negative bacterial proteins due to the lack of experimental data from Gram-positive bacteria (see above). In order to avoid potential errors, LocateP also scans all proteins assuming them to be Sec-secreted, except for the bacteriocin-like secreted proteins. If the Tat-secreted possibility score of a protein was significant, the final subcellular location of this protein was marked "Possibly Tat-" as an extra reference.

### Specific criteria for LPxTG-anchored and C-anchored protein prediction

The following topological criteria were used to identify LPxTG-type cell-wall anchored and C-anchored membrane proteins. For the selection of LPxTG-anchored proteins, the criteria were [165]: (i) the protein has only one N-terminal signal peptide/TM segment and only one C-terminal TM segment, (ii) the C-terminus of the protein contains an LPxTG-type motif; (iii) the LPxTG-type motif is followed by the C-terminal transmembrane helix and a positively charged C-terminal tail. These criteria were validated with 85 experimentally verified LPxTG-anchored proteins [166-171] and 83 of them were correctly identified.

The criteria used for predicting C-anchored proteins were: (i) the protein has only 2 predicted TM helices, one situated at the N-terminus and one at the C-terminus, (ii) the protein has a cleaved N-terminal signal peptide, (iii) the protein has a C-terminal transmembrane helix and a positively charged C-terminal tail but no LPxTG motif, (iv) the distance between the N-terminal and C-terminal helices is larger than 45 residues.

## Authors' contributions

RS conceived the project and acquired the funding, MM performed the research and constructed the LocateP pipeline and database, JB helped in tool selection, CF and RS supervised the project, while MM, CF and RS wrote the manuscript. All authors read and approved the manuscript.

## Supplementary Material

Additional file 1 **Flow chart and decision tree of the LocateP pipeline**. The different SCL tools used at each decision step are indicated. The different SCLs distinguished are boxed in the middle; "Bacteriocin" signifies bacteriocin-like proteins secreted by non-classical pathways, identified by Bagel. a) all tools agreed, b) all possible hits, c) majority vote, d) 2–3 TM segments and C-terminus detected by LPxTG HMM. Click here for file

Additional file 2 The LocateP predicted N-anchored and secreted proteins with known function in *Bacillus subtilis*. Click here for file

Additional file 3 The LocateP predicted N-anchored and secreted proteins of unknown function in *Bacillus subtilis*. Click here for file

Additional file 4 Literature references for other experimental evidence (Yes (O)) listed in additional file 2. Click here for file
