# Supplementary figures and images for: LocateP: Genome-scale subcellular-location predictor for bacterial proteins
Source: BMC Bioinformatics. 2008 Mar 27;9:173. doi: 10.1186/1471-2105-9-173 (PMC2375117; doi:10.1186/1471-2105-9-173)

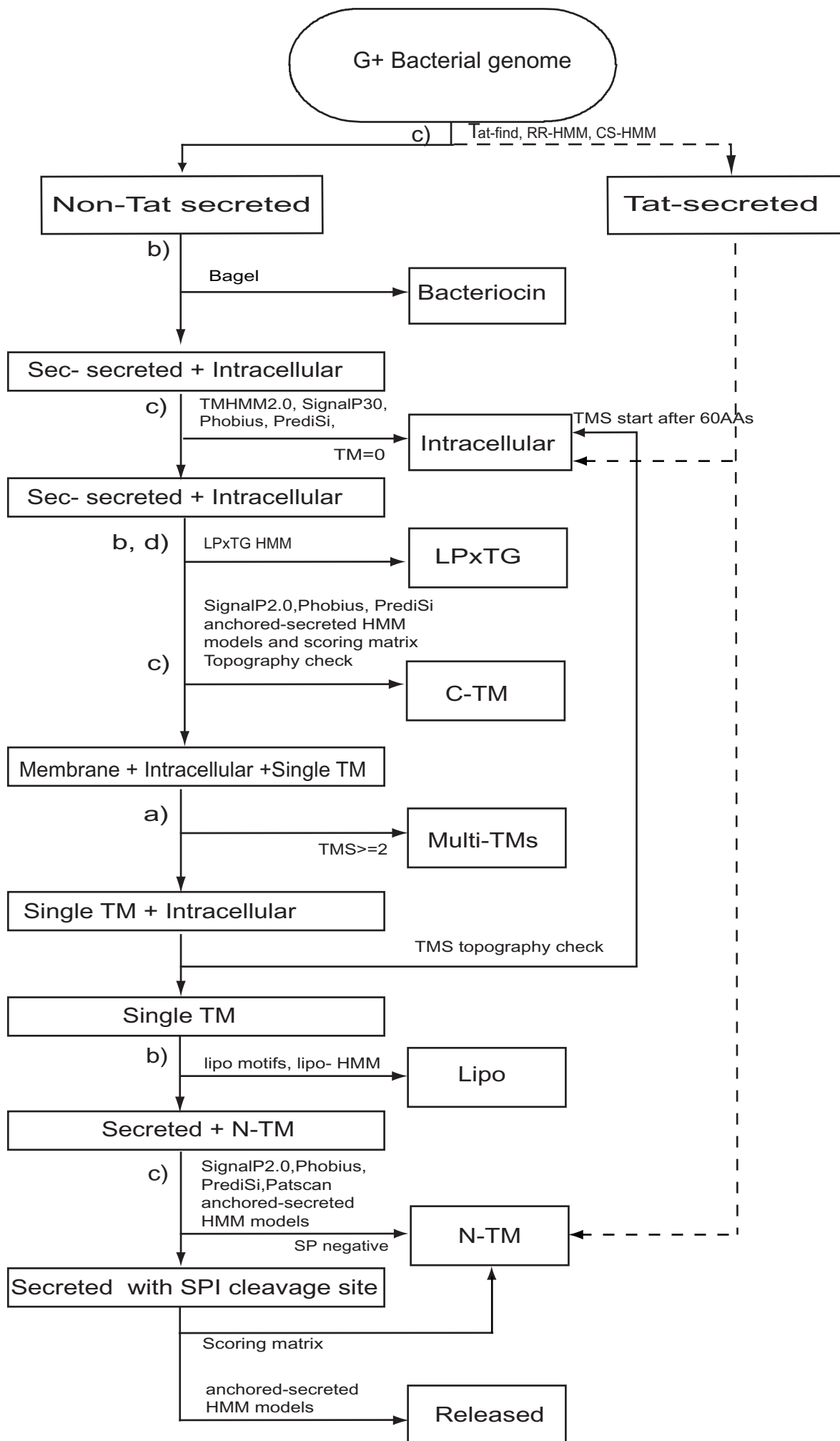

Supplement: Additional file 1 — Flow chart and decision tree of the LocateP pipeline. The different SCL tools used at each decision step are indicated. The different SCLs distinguished are boxed in the middle; "Bacteriocin" signifies bacteriocin-like proteins secreted by non-classical pathways, identified by Bagel. a) all tools agreed, b) all possible hits, c) majority vote, d) 2–3 TM segments and C-terminus detected by LPxTG HMM. [file 1471-2105-9-173-S1.pdf]
