# Supplementary material for: LocateP: Genome-scale subcellular-location predictor for bacterial proteins
Source: BMC Bioinformatics. 2008 Mar 27;9:173. doi: 10.1186/1471-2105-9-173 (PMC2375117; doi:10.1186/1471-2105-9-173)
Supplement: Additional file 2 — The LocateP predicted N-anchored and secreted proteins with known function in Bacillus subtilis. [file 1471-2105-9-173-S2.doc]

**Additional file 2.**

**Functionality/annotation of predicted N-anchored proteins of *Bacillus subtilis***

| ***Annotated protein function*** | ***gene**** | ***exp. verified*** | ***comment*** |
| --- | --- | --- | --- |
| **LOCATE-P PREDICTED N-ANCHORED PROTEINS** | | | |
| **Cell division** | | | |
| cell-division initiation protein | divIC | Yes (T) |  |
| cell-division initiation protein | divIB | Yes (O) |  |
| cell-division protein | ftsL | Yes (O) |  |
| cell-shape determining protein | mreC | Yes (T) |  |
| septation ring formation regulator EzrA | ezrA | Yes (O) | N_out_-C_in_ |
| **Cell wall biogenesis** | | | |
| biosynthesis of teichuronic acid | tuaA |  |  |
| D-alanine esterification of lipoteichoic acid and wall teichoic acid | dltD | Yes (T) |  |
| D-alanyl-D-alanine carboxypeptidase (penicillin-binding protein 5) | dacA* | Yes (T) |  |
| D-alanyl-D-alanine carboxypeptidase (penicillin-binding protein 5) | dacB* | Yes (T) |  |
| penicillin-binding protein (D-alanyl-D-alanine carboxypeptidase) | dacC | Yes (O) |  |
| penicilin binding protein (putative D-alanyl-D-alanine carboxypeptidase) | dacF* | Yes (T) |  |
| penicillin-binding proteins 1A and 1B | ponA* | Yes (T) |  |
| penicillin-binding protein 2A | pbpA |  |  |
| penicillin-binding protein 2B | pbpB | Yes (T) |  |
| penicillin-binding protein 2C | pbpF |  |  |
| penicillin-binding protein 4 | pbpD | Yes (T) |  |
| penicillin-binding protein | pbpX | Yes (O) |  |
| penicillin-binding protein | spoVD* | Yes (T) |  |
| polyglycerol phosphate assembly and export (teichoic acid biosynthesis) | tagB | Yes (O) |  |
| UTP-glucose-1-phosphate uridylyltransferase | gtaB | Yes (O) |  |
| **Cell wall turnover** | | | |
| cell wall hydrolase; phosphatase-associated protein (major autolysin) | lytE | Yes (T) |  |
| gamma-D-glutamate-meso-diaminopimelate muropeptidase (major autolysin) | lytF | Yes (T) |  |
| modifier protein of major autolysin LytC | lytB |  |  |
| N-acetylmuramoyl-L-alanine amidase | cwlD | Yes (O) |  |
| N-acetylmuramoyl-L-alanine amidase (major autolysin) | lytC* | Yes (T) |  |
| **Sporulation, germination** | | | |
| germination response to glucose, fructose, L-asparagine, and KCl | gerBC | Yes (O) |  |
| mutants block sporulation after engulfment (stage III sporulation) | spoIIIAH | Yes (T) |  |
| mutants block sporulation after engulfment (stage III sporulation) | spoIIIAG |  |  |
| required for completion of engulfment | spoIIQ | Yes (T) |  |
| required for dissolution of the septal cell wall (stage II sporulation) | spoIIP | Yes (T) |  |
| required for processing of pro-sigma-E (stage II sporulation) | spoIIR | Yes (O) |  |
| serine peptidase of the SA clan | spoIVB | Yes (T) |  |
| spore coat protein (outer) | cotC* | Yes (T) |  |
| spore cortex-lytic enzyme | sleB | Yes (O) |  |
| stage III sporulation protein SpoAB | spoIIIAB |  |  |
| forespore regulator of the sigma-K checkpoint | bofC | Yes (O) |  |
| **Motility** | | | |
| flagellar basal body-associated protein | fliL* | Yes (T) |  |
| flagellar motor protein | motB | Yes (O) |  |
| flagellar motor protein | ytxE |  |  |
| **Transport, translocation** | | | |
| component of the twin-arginine pre-protein translocation pathway | tatAY |  |  |
| component of the twin-arginine pre-protein translocation pathway | tatAD |  |  |
| preprotein translocase subunit | secE | Yes (T) |  |
| preprotein translocase subunit YajC | yajC | Yes (O) |  |
| phosphotransferase system (PTS) lichenan-specific enzyme IIB | licB | Yes (O) |  |
| holin-like protein | bhlA |  |  |
| type I signal peptidase | sipT | Yes (T) |  |
| type I signal peptidase | sipU | Yes (T) |  |
| type I signal peptidase | sipS | Yes (O) |  |
| type I signal peptidase | sipV | Yes (O) |  |
| possible C4-dicarboxylate binding protein | dctB | Yes (O) |  |
| **Competence** | | | |
| exogenous DNA-binding protein | comGC | Yes (O) |  |
| probably part of the DNA transport machinery | comGD | Yes (O) |  |
| probably part of the DNA transport machinery | comGE | Yes (O) |  |
| probably part of the DNA transport machinery | comGG | Yes (O) |  |
| unspecific high-affinity DNA-binding protein | comEA | Yes (O) |  |
| extracellular DNase | nucB | Yes (O) |  |
| membrane-associated DNase | nucA | Yes (O) |  |
| **Regulation** | | | |
| membrane-bound transcriptional regulator LytR | lytR | Yes (T) |  |
| negative regulation of sigma-X activity | rsiX | Yes (O) |  |
| inhibitor of the activity of phosphatase RapA | phrA | Yes (O) | precursor of extracellular quorum-sensing peptide |
| regulator of the activity of phosphatase RapE | phrE | Yes (O) | precursor of extracellular quorum-sensing peptide |
| regulator of the activity of phosphatase RapF | phrF | Yes (O) | precursor of extracellular quorum-sensing peptide |
| regulator of the activity of phosphatase RapG | phrG | Yes (O) | precursor of extracellular quorum-sensing peptide |
| regulator of the activity of phosphatase RapI | phrI | Yes (O) | precursor of extracellular quorum-sensing peptide |
| regulator of the activity of phosphatase RapK | phrK | Yes (O) | precursor of extracellular quorum-sensing peptide |
| transcriptional regulator (IclR family) | kipR |  |  |
| **Miscellaneous: other functions** | | | |
| ATP synthase subunit b | atpF | Yes (T) |  |
| carboxy-terminal processing protease | ctpA* | Yes (T) |  |
| Lon-like ATP-dependent protease | lonB | Yes (O) |  |
| serine protease Do (heat-shock protein) | htrA | Yes (T) |  |
| pectate lyase | pel |  |  |
| pectate lyase | pelB |  |  |
| cytochrome c550 | cccA | Yes (T) |  |
| menaquinol:cytochrome c oxidoreductase (iron-sulfur subunit) | qcrA |  |  |
| protoporphyrinogen oxidase | hemY |  |  |
| succinate dehydrogenase | sdhA* | Yes (T) | N_out_-C_in_ |
| thiol-disulfide oxidoreductase | bdbA | Yes (T) |  |
| NAD(P)H-dependent glycerol-3-phosphate dehydrogenase | gpsA | Yes (O) |  |
| 44 hypothetical proteins (unknown function) | (21)* | Yes (T) |  |
| 122 hypothetical proteins (unknown function) |  |  |  |
| **Uncertain if N-anchored** | | | |
| 3-ketoacyl-(acyl-carrier-protein) reductase | fabG |  | fatty acid biosynthesis |
| 6-phospho-alpha-glucosidase | malA |  | maltose metabolism and transport ? |
| antilisterial bacteriocin (subtilosin) production | albE |  | biosynthesis of exported bacteriocin |
| glucose-1-phosphate adenylyltransferase | glgC |  | glycogen biosynthesis |
| short chain dehydrogenase | yxbG |  |  |
| **Incorrectly predicted N-anchored proteins** | | | |
| glycine oxidase | goxB |  | intracellular |
| L-asparaginase | ansA |  | intracellular |
| L-lactate dehydrogenase | ldh |  | intracellular |
| malate dehydrogenase | mdh | Yes (O) | intracellular |
| pyrroline-5-carboxylate reductase | proH |  | intracellular |
| prephenate dehydrogenase | tyrA |  | intracellular |
| modification methylase Bsu (restriction-modification) | mtbP |  | intracellular |

**Functionality/annotation of predicted secreted proteins of *Bacillus subtilis***

| **LOCATE-P PREDICTED SECRETED PROTEINS** | | | |
| --- | --- | --- | --- |
| **Extracellular proteases/peptidases** | | | |
| bacillopeptidase F | bpr* | Yes (T) |  |
| extracellular metalloprotease | mpr* | Yes (T) |  |
| extracellular neutral metalloprotease | nprE* | Yes (T) |  |
| extracellular neutral protease B | nprB | Yes (O) |  |
| extracellular serine protease | vpr* | Yes (T) |  |
| extracellular serine protease | epr | Yes (O) |  |
| gamma-glutamyltranspeptidase | ggt* | Yes (T) |  |
| serine alkaline protease (subtilisin E) | aprE* | Yes (T) |  |
| **Extracellular carbohydrases** | | | |
| alpha-amylase | amyE* | Yes (T) |  |
| arabinan-endo 1,5-alpha-L-arabinase | abnA* | Yes (T) |  |
| chitosanase | csn* | Yes (T) |  |
| endo-1,4-beta-glucanase | bglC* | Yes (T) |  |
| endo-1,4-beta-xylanase | xynA* | Yes (T) |  |
| endo-1,4-beta-xylanase (xylanase D) | xynD* | Yes (T) |  |
| endo-beta-1,3-1,4 glucanase | bglS* | Yes (T) |  |
| levanase | sacC | Yes (O) |  |
| levansucrase | sacB | Yes (O) |  |
| **Miscellaneous: enzymes** | | | |
| alkaline phosphatase A | phoA | Yes (O) |  |
| alkaline phosphatase III | phoB | Yes (O) |  |
| beta-lactamase precursor | penP |  |  |
| extracellular esterase | lipB | Yes (O) |  |
| phytase | phy |  |  |
| glycerophosphoryl diester phosphodiesterase | glpQ | Yes (O) |  |
| **Miscellaneous: other functions** | | | |
| cell wall-associated protein precursor | wapA | Yes (O) |  |
| cell wall-associated protein precursor | wprA | Yes (O) |  |
| component of the twin-arginine pre-protein translocation pathway | tatAC | Yes (O) |  |
| translocation-dependent antimicrobial spore component | tasA* | Yes (T) |  |
| 21 hypothetical proteins (unknown function) | (21)* | Yes (T) |  |
| 45 hypothetical proteins (unknown function) |  |  |  |
| **Uncertain if secreted** | | | |
| regulator of the activity of phosphatase RapC and competence and sporulation stimulating factor (CSF) | phrC |  | precursor of extracellular quorum-sensing peptide |
| required for complete dissolution of the asymmetric septum | spoIID |  |  |
| short chain dehydrogenase | yusZ |  |  |
| **Incorrectly predicted secreted proteins** | | | |
| aspartate aminotransferase | aspB |  | intracellular |
| L-aspartate oxidase | nadB |  | intracellular |
| N-acetylglucosaminidase (major autolysin) | lytD | Yes (O) | extracellular N-anchored ? |
| signal peptide peptidase | sppA | Yes (O) | membrane associated |
| multiple sugar-binding protein | msmE |  | extracellular lipoprotein |
| thiol-disulfide oxidoreductase | bdbD | Yes (T) | N-anchored protein |
| hypothetical protein | ykvT | Yes (T) | N-anchored protein |
| hypothetical protein | yvpB | Yes (T) | N-anchored protein |
| hypothetical protein | ydjN | Yes (T) | N-anchored protein |
| hypothetical protein | yqzC | Yes (T) | N-anchored protein |
| hypothetical protein | ypmS | Yes (T) | N-anchored protein |

Yes (T), the proteins are experimentally verified by Tjalsma *et al* [1]; there are 36 secreted, and 30 N-anchored proteins (YtwP was not found in the annotated *B.subtilis* genome sequence) containing a putative SPIase-cleavage site and 48 N-anchored proteins containing no putative SPI-cleavage site;

Yes (O): the location of the protein is experimentally verified in other studies (Literature references in Additional file 4).

*: the proteins have a putative SPI-cleavage site and are included in the LocateP training set to distinguish N-anchored and secreted proteins.

N_out_-C_in_: the C-terminus of the protein is at the cytoplasmic side whereas the N-terminus is at the outer side of the bacterial cell membrane.

1. Tjalsma H, van Dijl JM**: Proteomics-based consensus prediction of protein retention in a bacterial membra**ne*. Proteomic*s 2005**,** 5(17):4472-4482.
