# Supplementary material for: LocateP: Genome-scale subcellular-location predictor for bacterial proteins
Source: BMC Bioinformatics. 2008 Mar 27;9:173. doi: 10.1186/1471-2105-9-173 (PMC2375117; doi:10.1186/1471-2105-9-173)
Supplement: Additional file 4 — Literature references for other experimental evidence (Yes (O)) listed in additional file 2. [file 1471-2105-9-173-S4.doc]

# Literature references of additional file 2

1. Antelmann H, Towe S, Albrecht D, Hecker M: **The phosphorus source phytate changes the composition of the cell wall proteome in Bacillus subtilis**. *J Proteome Res* 2007, **6**(2):897-903.

2. Aung S, Shum J, Abanes-De Mello A, Broder DH, Fredlund-Gutierrez J, Chiba S, Pogliano K: **Dual localization pathways for the engulfment proteins during Bacillus subtilis sporulation**. *Mol Microbiol* 2007, **65**(6):1534-1546.

3. Lanigan-Gerdes S, Dooley AN, Faull KF, Lazazzera BA: **Identification of subtilisin, Epr and Vpr as enzymes that produce CSF, an extracellular signalling peptide of Bacillus subtilis**. *Mol Microbiol* 2007, **65**(5):1321-1333.

4. Bhavsar AP, D'Elia MA, Sahakian TD, Brown ED: **The Amino Terminus of Bacillus subtilis TagB Possesses Separable Localization and Functional Properties**. *J Bacteriol* 2007, **189**(19):6816-6823.

5. Singh JK, Makde RD, Kumar V, Panda D: **A Membrane Protein, EzrA, Regulates Assembly Dynamics of FtsZ by Interacting with the C-Terminal Tail of FtsZ**. *Biochemistry* 2007, **46**(38):11013-11022.

6. Schreiber S, Stengel R, Westermann M, Volkmer-Engert R, Pop OI, Muller JP: **Affinity of TatCd for TatAd elucidates its receptor function in the Bacillus subtilis twin arginine translocation (Tat) translocase system**. *J Biol Chem* 2006, **281**(29):19977-19984.

7. Rotanova TV, Botos I, Melnikov EE, Rasulova F, Gustchina A, Maurizi MR, Wlodawer A: **Slicing a protease: structural features of the ATP-dependent Lon proteases gleaned from investigations of isolated domains**. *Protein Sci* 2006, **15**(8):1815-1828.

8. Masayama A, Fukuoka H, Kato S, Yoshimura T, Moriyama M, Moriyama R: **Subcellular localization of a germiantion-specific cortex-lytic enzyme, SleB, of Bacilli during sporulation**. *Genes Genet Syst* 2006, **81**(3):163-169.

9. Daniel RA, Noirot-Gros MF, Noirot P, Errington J: **Multiple interactions between the transmembrane division proteins of Bacillus subtilis and the role of FtsL instability in divisome assembly**. *J Bacteriol* 2006, **188**(21):7396-7404.

10. Thompson LS, Beech PL, Real G, Henriques AO, Harry EJ: **Requirement for the cell division protein DivIB in polar cell division and engulfment during sporulation in Bacillus subtilis**. *J Bacteriol* 2006, **188**(21):7677-7685.

11. Doan T, Marquis KA, Rudner DZ: **Subcellular localization of a sporulation membrane protein is achieved through a network of interactions along and across the septum**. *Mol Microbiol* 2005, **55**(6):1767-1781.

12. Scheffers DJ: **Dynamic localization of penicillin-binding proteins during spore development in Bacillus subtilis**. *Microbiology* 2005, **151**(Pt 3):999-1012.

13. Noirclerc-Savoye M, Le Gouellec A, Morlot C, Dideberg O, Vernet T, Zapun A: **In vitro reconstitution of a trimeric complex of DivIB, DivIC and FtsL, and their transient co-localization at the division site in Streptococcus pneumoniae**. *Mol Microbiol* 2005, **55**(2):413-424.

14. Patterson HM, Brannigan JA, Cutting SM, Wilson KS, Wilkinson AJ, Ab E, Diercks T, de Jong RN, Truffault V, Folkers GE *et al*: **The structure of bypass of forespore C, an intercompartmental signaling factor during sporulation in Bacillus**. *J Biol Chem* 2005, **280**(43):36214-36220.

15. Serrano M, Neves A, Soares CM, Moran CP, Jr., Henriques AO: **Role of the anti-sigma factor SpoIIAB in regulation of sigmaG during Bacillus subtilis sporulation**. *J Bacteriol* 2004, **186**(12):4000-4013.

16. Rubio A, Pogliano K: **Septal localization of forespore membrane proteins during engulfment in Bacillus subtilis**. *Embo J* 2004, **23**(7):1636-1646.

17. Haeusser DP, Schwartz RL, Smith AM, Oates ME, Levin PA: **EzrA prevents aberrant cell division by modulating assembly of the cytoskeletal protein FtsZ**. *Mol Microbiol* 2004, **52**(3):801-814.

18. Sanyal SC, Pal S, Chowdhury S, DasGupta C: **23S rRNA assisted folding of cytoplasmic malate dehydrogenase is distinctly different from its self-folding**. *Nucleic Acids Res* 2002, **30**(11):2390-2397.

19. Chirakkal H, O'Rourke M, Atrih A, Foster SJ, Moir A: **Analysis of spore cortex lytic enzymes and related proteins in Bacillus subtilis endospore germination**. *Microbiology* 2002, **148**(Pt 8):2383-2392.

20. Hudson KD, Corfe BM, Kemp EH, Feavers IM, Coote PJ, Moir A: **Localization of GerAA and GerAC germination proteins in the Bacillus subtilis spore**. *J Bacteriol* 2001, **183**(14):4317-4322.

21. Eggert T, Pencreac'h G, Douchet I, Verger R, Jaeger KE: **A novel extracellular esterase from Bacillus subtilis and its conversion to a monoacylglycerol hydrolase**. *Eur J Biochem* 2000, **267**(21):6459-6469.

22. Antelmann H, Scharf C, Hecker M: **Phosphate starvation-inducible proteins of Bacillus subtilis: proteomics and transcriptional analysis**. *J Bacteriol* 2000, **182**(16):4478-4490.

23. Tortosa P, Albano M, Dubnau D: **Characterization of ylbF, a new gene involved in competence development and sporulation in Bacillus subtilis**. *Mol Microbiol* 2000, **35**(5):1110-1119.

24. Sievers J, Errington J: **The Bacillus subtilis cell division protein FtsL localizes to sites of septation and interacts with DivIC**. *Mol Microbiol* 2000, **36**(4):846-855.

25. Katis VL, Wake RG, Harry EJ: **Septal localization of the membrane-bound division proteins of Bacillus subtilis DivIB and DivIC is codependent only at high temperatures and requires FtsZ**. *J Bacteriol* 2000, **182**(12):3607-3611.

26. Stover AG, Driks A: **Regulation of synthesis of the Bacillus subtilis transition-phase, spore-associated antibacterial protein TasA**. *J Bacteriol* 1999, **181**(17):5476-5481.

27. Bolhuis A, Matzen A, Hyyrylainen HL, Kontinen VP, Meima R, Chapuis J, Venema G, Bron S, Freudl R, van Dijl JM: **Signal peptide peptidase- and ClpP-like proteins of Bacillus subtilis required for efficient translocation and processing of secretory proteins**. *J Biol Chem* 1999, **274**(35):24585-24592.

28. Leloup L, Le Saux J, Petit-Glatron MF, Chambert R: **Kinetics of the secretion of Bacillus subtilis levanase overproduced during the exponential phase of growth**. *Microbiology* 1999, **145 (Pt 3)**:613-619.

29. Margot P, Pagni M, Karamata D: **Bacillus subtilis 168 gene lytF encodes a gamma-D-glutamate-meso-diaminopimelate muropeptidase expressed by the alternative vegetative sigma factor, sigmaD**. *Microbiology* 1999, **145 (Pt 1)**:57-65.

30. Kato J, Nakamura T, Kuroda A, Ohtake H: **Cloning and characterization of chemotaxis genes in Pseudomonas aeruginosa**. *Biosci Biotechnol Biochem* 1999, **63**(1):155-161.

31. Stamm LV, Bergen HL: **Molecular characterization of a flagellar (fla) operon in the oral spirochete Treponema denticola ATCC 35405**. *FEMS Microbiol Lett* 1999, **179**(1):31-36.

32. Katis VL, Wake RG: **Membrane-bound division proteins DivIB and DivIC of Bacillus subtilis function solely through their external domains in both vegetative and sporulation division**. *J Bacteriol* 1999, **181**(9):2710-2718.

33. Chung YS, Breidt F, Dubnau D: **Cell surface localization and processing of the ComG proteins, required for DNA binding during transformation of Bacillus subtilis**. *Mol Microbiol* 1998, **29**(3):905-913.

34. Pedersen LB, Murray T, Popham DL, Setlow P: **Characterization of dacC, which encodes a new low-molecular-weight penicillin-binding protein in Bacillus subtilis**. *J Bacteriol* 1998, **180**(18):4967-4973.

35. Daniel RA, Harry EJ, Katis VL, Wake RG, Errington J: **Characterization of the essential cell division gene ftsL(yIID) of Bacillus subtilis and its role in the assembly of the division apparatus**. *Mol Microbiol* 1998, **29**(2):593-604.

36. Lunsford RD, Roble AG: **comYA, a gene similar to comGA of Bacillus subtilis, is essential for competence-factor-dependent DNA transformation in Streptococcus gordonii**. *J Bacteriol* 1997, **179**(10):3122-3126.

37. Katis VL, Harry EJ, Wake RG: **The Bacillus subtilis division protein DivIC is a highly abundant membrane-bound protein that localizes to the division site**. *Mol Microbiol* 1997, **26**(5):1047-1055.

38. Morbidoni HR, de Mendoza D, Cronan JE, Jr.: **Synthesis of sn-glycerol 3-phosphate, a key precursor of membrane lipids, in Bacillus subtilis**. *J Bacteriol* 1995, **177**(20):5899-5905.

39. Inamine GS, Dubnau D: **ComEA, a Bacillus subtilis integral membrane protein required for genetic transformation, is needed for both DNA binding and transport**. *J Bacteriol* 1995, **177**(11):3045-3051.

40. van Sinderen D, Kiewiet R, Venema G: **Differential expression of two closely related deoxyribonuclease genes, nucA and nucB, in Bacillus subtilis**. *Mol Microbiol* 1995, **15**(2):213-223.

41. Chung YS, Dubnau D: **ComC is required for the processing and translocation of comGC, a pilin-like competence protein of Bacillus subtilis**. *Mol Microbiol* 1995, **15**(3):543-551.

42. Hulett FM, Lee J, Shi L, Sun G, Chesnut R, Sharkova E, Duggan MF, Kapp N: **Sequential action of two-component genetic switches regulates the PHO regulon in Bacillus subtilis**. *J Bacteriol* 1994, **176**(5):1348-1358.

43. Pogliano KJ, Beckwith J: **Genetic and molecular characterization of the Escherichia coli secD operon and its products**. *J Bacteriol* 1994, **176**(3):804-814.

44. McCarter LL: **MotY, a component of the sodium-type flagellar motor**. *J Bacteriol* 1994, **176**(14):4219-4225.

45. Levin PA, Losick R: **Characterization of a cell division gene from Bacillus subtilis that is required for vegetative and sporulation septum formation**. *J Bacteriol* 1994, **176**(5):1451-1459.

46. Harry EJ, Stewart BJ, Wake RG: **Characterization of mutations in divIB of Bacillus subtilis and cellular localization of the DivIB protein**. *Mol Microbiol* 1993, **7**(4):611-621.

47. Laible G, Keck W, Lurz R, Mottl H, Frere JM, Jamin M, Hakenbeck R: **Penicillin-binding protein 2x of Streptococcus pneumoniae. Expression in Escherichia coli and purification of a soluble enzymatically active derivative**. *Eur J Biochem* 1992, **207**(3):943-949.

48. Tran L, Wu XC, Wong SL: **Cloning and expression of a novel protease gene encoding an extracellular neutral protease from Bacillus subtilis**. *J Bacteriol* 1991, **173**(20):6364-6372.

49. Estrela AI, Pooley HM, de Lencastre H, Karamata D: **Genetic and biochemical characterization of Bacillus subtilis 168 mutants specifically blocked in the synthesis of the teichoic acid poly(3-O-beta-D-glucopyranosyl-N-acetylgalactosamine 1-phosphate): gneA, a new locus, is associated with UDP-N-acetylglucosamine 4-epimerase activity**. *J Gen Microbiol* 1991, **137**(4):943-950.

50. Laible G, Hakenbeck R, Sicard MA, Joris B, Ghuysen JM: **Nucleotide sequences of the pbpX genes encoding the penicillin-binding proteins 2x from Streptococcus pneumoniae R6 and a cefotaxime-resistant mutant, C506**. *Mol Microbiol* 1989, **3**(10):1337-1348.
